# Aquaporin‐1 differentiates intrahepatic cholangiocarcinoma from liver metastases of pancreatic ductal adenocarcinoma

**DOI:** 10.1111/his.70108

**Published:** 2026-01-29

**Authors:** Leoncini Giuseppe, Barella Marco, Droz Dit Busset Michele, Gentili Marco, Bodini Beatrice, Ronchetti Simona, Cari Luigi, Mazzaferro Vincenzo

**Affiliations:** ^1^ First Pathology Division, Department of Services and Advanced Diagnostics Fondazione IRCCS Istituto Nazionale dei Tumori Milan Italy; ^2^ HPB Surgery, Hepatology and Liver Transplantation Unit Fondazione IRCCS Istituto Nazionale Tumori Milan Italy; ^3^ Tumor Genomics Unit, Department of Research Fondazione IRCCS Istituto Nazionale dei Tumori Milan Italy; ^4^ Pharmacology Division, Department of Medicine and Surgery University of Perugia Perugia Italy; ^5^ Department of Oncology and Hemato‐Oncology University of Milan Milan Italy

**Keywords:** aquaporin‐1, differential diagnosis, intrahepatic cholangiocarcinoma, liver metastases, pancreatic ductal adenocarcinoma

## Abstract

**Aims:**

Differentiating intrahepatic cholangiocarcinoma (iCCA) from metastatic ductal adenocarcinoma of the pancreas (mPDAC) is challenging due to commonalities in histology and phenotype. Currently, available biomarkers are not completely satisfactory in supporting such differential diagnosis. The present study aims to evaluate aquaporin (AQP)‐1 as a reliable biliary biomarker.

**Methods and results:**

A total of 151 pancreatobiliary adenocarcinomas, including 77 surgical resections and 74 needle biopsies, were considered, including 84 CCA and 67 PDAC. Immunohistochemistry was performed in two steps: (1) primary tumours, including CCA and PDAC, were compared; (2) data from primary tumours were subsequently validated on needle biopsies, comparing iCCA and mPDAC. The staining was examined according to the immunoreactive score (IRS). On surgical resections, our evaluation showed a mean IRS value of 11.07 in iCCA and 0.7 in PDAC; likewise, on needle biopsies, a mean IRS value of 10.54 in iCCA and 0.97 in mPDAC was observed. Combining surgical resections and needle biopsies, the mean IRS value resulted in 10.76 in iCCA; conversely, it was 0.84 in PDAC. Overall, the ROC curves showed the AQP1 diagnostic performance to be characterized by an AUC of 0.999, being a sensitivity of 100% and a specificity of 95.45%.

**Conclusions:**

AQP1 is a novel biliary biomarker which has been shown to outperform other biliary biomarkers. The main diagnostic scenario in which AQP1 staining should be used is to distinguish iCCA from mPDAC. Furthermore, differential diagnosis between eCCA and PDAC in surgical specimens and between PDAC and CP in needle biopsies should be considered.

## Graphical abstract

Differentiating intrahepatic CCA from PDAC metastatic to the liver is critical for the clinical management of patients with pancreatobiliary cancers. AQP1 has demonstrated an excellent discriminatory performance in this clinical scenario.

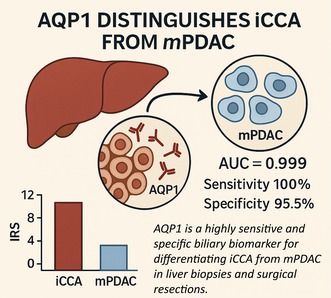

**Abbreviations**
AQPs: aquaporins
AUC: area under the curve
CCA: cholangiocarcinoma
CP: chronic pancreatitis
CRP: C‐reactive protein
HCC: hepatocellular carcinoma
iCCA: intrahepatic cholangiocarcinoma
IPMN: intraductal papillary mucinous neoplasm
IPNB: intraductal papillary neoplasm of the bile duct
IRS: immunoreactive score
ISH: in situ hybridization
MCN: mucinous cystic neoplasm
mPDAC: metastatic ductal adenocarcinoma of the pancreas
PanIN: pancreatic intraepithelial neoplasia
ROC: receiver operating characteristic

## Introduction

Pancreatobiliary cancers are aggressive tumours with common embryogenesis, histological features and phenotypes.^1^ Cholangiocarcinoma (CCA) is classified into intrahepatic (iCCA) and extrahepatic (eCCA), the latter being further subdivided into peri‐hilar (pCCA) and distal (dCCA).^2^ They display three main types of growth, including mass‐forming, periductal infiltrating and intraductal patterns.^3^ PDAC is the fourth leading cause of cancer‐related death.^4^ Established precursor lesions are pancreatic intraepithelial neoplasia (PanIN), intraductal papillary mucinous neoplasm (IPMN) and mucinous cystic neoplasm (MCN). It is characterized by aggressive behaviour and a poor prognosis. Liver metastases impact dramatically on the long‐term survival of PDAC patients.^5^, ^6^, ^7^ The broad array of similarities poses diagnostic challenges in distinguishing iCCA from mPDAC in the liver. In addition, the distinction between eCCA and PDAC cannot be based on histology alone, and further investigation is required.

Histological diagnosis informs clinical management. The mPDAC is treated with palliative chemotherapy protocols, whereas treatment options for iCCA include surgical resection, neoadjuvant chemotherapy or locoregional therapies.^6^, ^8^ Diagnostic liver biopsy is generally recommended, but in most cases, the diagnostic decision relies on supportive information obtained from imaging. However, current radiological methods, especially CT scan, are unable to differentiate iCCA from mPDAC and have suboptimal accuracy in identifying pancreatic lesions.^6^, ^8^ Currently, distinguishing iCCA from mPDAC on liver biopsies remains a challenging task for pathologists. Although genomic and epigenetic analyses have been proposed for improving diagnostic accuracy in pancreatobiliary malignancies, the costs are high compared to immunohistochemistry. Moreover, molecular assessments cannot be integrated into the routine diagnostic workflow.^9^, ^10^, ^11^

Aquaporins (AQPs) are membrane‐bound water‐specific channels that ensure efficient cell permeability. Water transport is a primary condition for homeostasis, which relies on diffusion movements in cells lacking water‐specific channels.^12^ Thirteen AQPs have been identified so far.^13^, ^14^ Water transport is a crucial step in many physiological processes, including bile formation, which represents a fundamental requirement for liver homeostasis.^15^, ^16^ Particularly, AQP1 is constitutively expressed in cholangiocytes, and its involvement in bile duct development has been established.^17^, ^18^

The present study aims to investigate the role of AQP1 in distinguishing iCCA from mPDAC.

## Materials and Methods

### Patient Selection

The study was designed as a retrospective, single‐centre analysis, focused on the evaluation of AQP1 protein expression in pancreatobiliary adenocarcinomas. The study was performed according to the clinical standards of the Declaration of Helsinki, and it was approved by the Ethics Committee of Fondazione IRCCS INT (No. INT 199/22). Specimens from 151 pancreatobiliary adenocarcinomas were retrieved from the institutional archive and histologically reassessed. CCA was classified into intrahepatic and extrahepatic, the former being further subdivided into small duct and large duct types, according to the WHO guidelines.^19^ The PDAC included the three grades of differentiation, from well to poorly differentiated adenocarcinomas. The case series included both CCA and PDAC, consisting of 77 surgical resections and 74 needle biopsies. The surgical specimens were evaluated for AQP1 expression in primary CCA and PDAC. Biopsies for AQP1 expression in iCCA and mPDAC. Both biliary and pancreatic preinvasive lesions were included in our investigation. Clinical–pathological data were also collected (Tables S1 and S2). Imaging validation was warranted for mPDAC on liver biopsies.

### Immunohistochemical Evaluation

Specimens were cut into 4‐micron‐thick slides, deparaffinized and reacted with monoclonal antibody against AQP1 (clone *1/22*; dilution 1:1000; AbCAM, Cambridge, UK). Immunohistochemical staining was evaluated by two expert pathologists (GL, MB), and membranous immunolabelling was considered positive, according to the expression pattern shown by the internal control (bile and lobular pancreatic ducts). An immunoreactive score (IRS) was obtained for each case, and two parameters were investigated: (1) the percentage of positive neoplastic cells and (2) the staining intensity. The proportion of positive cells was evaluated as score 0 (no positive cells), score 1 (1%–5% positive cells), score 2 (6%–10% positive cells), score 3 (11%–50% positive cells) and score 4 (51%–100% positive cells). Intensity was evaluated as score 0 (undetectable staining), score 1 (weak staining), score 2 (moderate staining) or score 3 (strong staining). The final IRS score ranged from 0 to 12 and was obtained by multiplying the two parameters.^20^ A variable degree of heterogeneity was due to the evidence of cytoplasmic staining, but this qualitative variation was considered as non‐specific. For the scoring purposes, only the membranous AQP1 expression was considered, according to its expected functional location. In quantitative terms, we have considered the proportion of positive membranous staining compared to the whole tumour area in each field. The array of variability in AQP1 staining is reported in Figure S1.

### Statistical Analysis

The proportion of AQP1‐positive cancer cells and the staining intensity were assessed for each patient's subgroup. The frequencies of different staining intensities and expression profiles were then compared between subgroups. The contingency test (chi‐squared test) was used to evaluate statistical differences. Due to the non‐normal distribution of the data, as assessed by the Kolmogorov–Smirnov normality test, the Kruskal–Wallis test with uncorrected Dunn's post hoc test was used for comparisons between subgroups. Receiver operating characteristic (ROC) curve analysis (Wilson/Brown method) was employed to assess the diagnostic potential of AQP1 as a biliary biomarker. Statistical analysis was conducted using PRISM version 9.5.1 (GraphPad, Boston, MA, USA), with statistical significance set at *P* < 0.05.

## Results

Histological assessment showed 84 CCA cases, further subdivided into iCCA (no 66; no 34 small duct type: no 32 large duct type) and eCCA (no 18) and 67 PDAC cases. Particularly, the surgical specimens showed PDAC in 33 cases, iCCA in 27 cases, and eCCA in 17 cases, the latter consisting of 5 pCCA and 12 dCCA. Biopsy specimens were composed of 34 mPDAC and 39 iCCA; one dCCA case was also included. The gland‐forming pattern was found in 69 of 84 CCA cases (82%), and the solid pattern was found in 15 cases (18%).

### AQP1 Expression in Normal Liver and Pancreas

**Figure 1:**
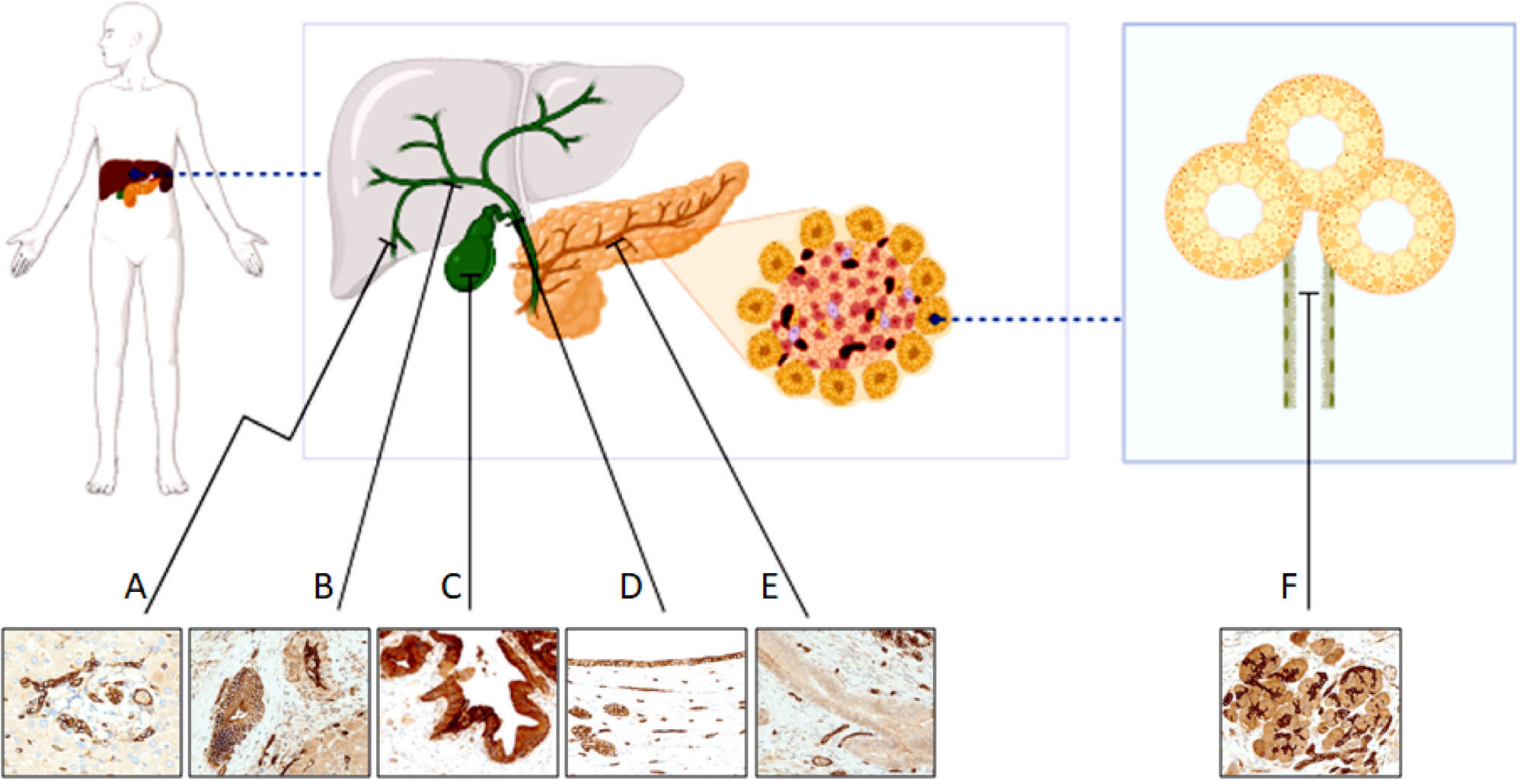
Pictorial representation of the AQP1 immunohistochemical expression in the normal pancreatobiliary system. Note that diffuse membranous expression is present in small intrahepatic bile ducts (diameter <60 μm, **A**), in medium‐ to large‐sized bile ducts (diameter >60 μm, **B**), as well as in gallbladder mucosa (**C**) and extrahepatic bile ducts (**D**). The pancreatic ducts do not express AQP1 (**E**), except for the lobular ducts (**F**).

The AQP1 expression in liver and pancreatic parenchyma was compared through immunohistochemical evaluation of perilesional areas. AQP1 was expressed in the biliary tree and the lobular pancreatic duct, showing membranous expression. Hepatocytes did not stain for AQP1. The AQP1 staining was restricted to the lobular ducts, whereas large‐sized ducts did not stain. In addition, acinic and insular compartments were non‐reactive (Figure 1). Endothelial cells lining both intrahepatic portal vein branches and centrilobular veins showed AQP1 staining. In contrast, hepatic sinusoids did not stain for AQP1. The endothelial lining also exhibited AQP1 expression in the pancreas. Furthermore, the peripheral nerves resulted in AQP1‐positive staining in the perineurium (Figure S2).

### AQP1 Expression in Pre‐Invasive Biliary and Pancreatic Tumours

**Figure 2:**
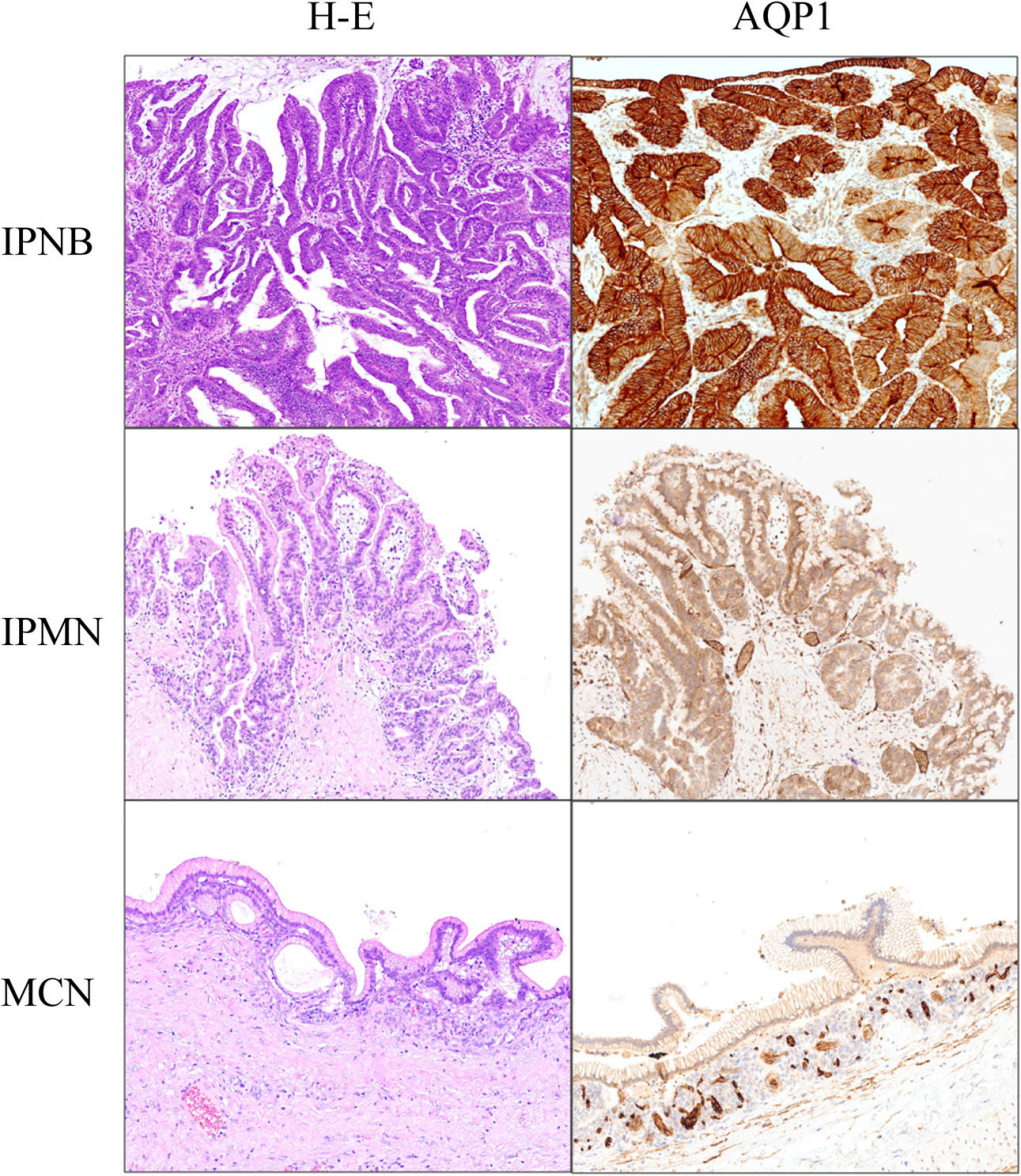
The AQP1 membranous expression characterizes pre‐invasive biliary neoplasms, including the intraductal papillary neoplasm of the bile duct (IPNB) (*upper panel*), whereas it was absent in both intraductal papillary mucinous neoplasm (IPMN) (*central panel*) and mucinous cystic neoplasm (MCN) of the pancreas (*lower panel*).

Pre‐invasive pancreatobiliary lesions were also evaluated for AQP1 expression. Nevertheless, the overall expression in pre‐invasive lesions mirrored that in liver and pancreas parenchyma, particularly intraductal papillary neoplasm of the bile ducts (IPNB) stains for AQP1. In contrast, IPMN, MCN, and PanIN did not stain (Figure 2).

### AQP1 Expression in CCA Versus Primary PDAC

**Figure 3:**
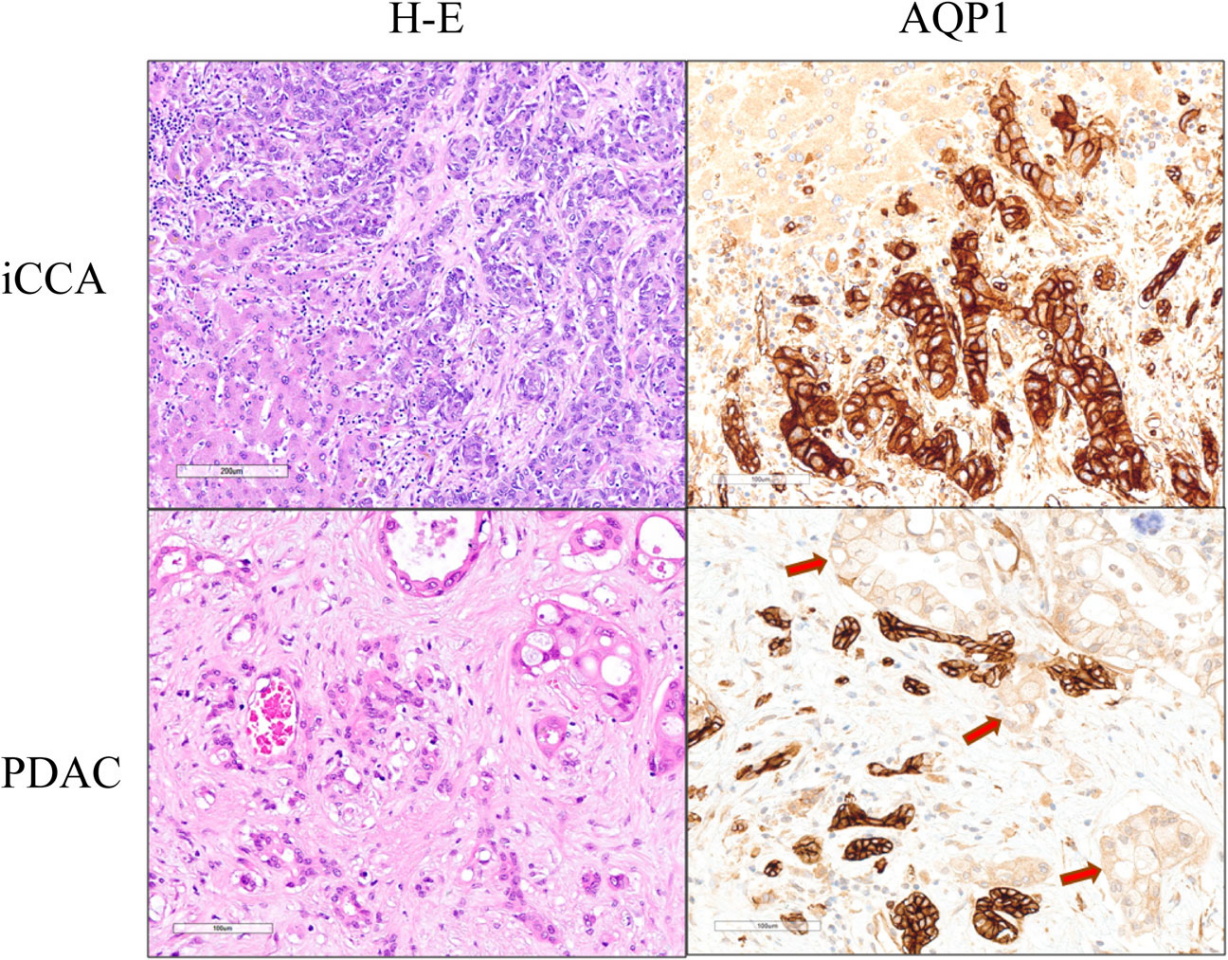
The AQP1 membranous expression in pancreatobiliary adenocarcinomas shows iCCA (*upper panel*) to be characterized by strong membranous expression, whereas ductal adenocarcinoma of the pancreas is negative (*lower panel*, *arrows*). Note that lobular ducts are present, showing AQP1 expression. [Colour figure can be viewed at wileyonlinelibrary.com]

**Figure 4:**
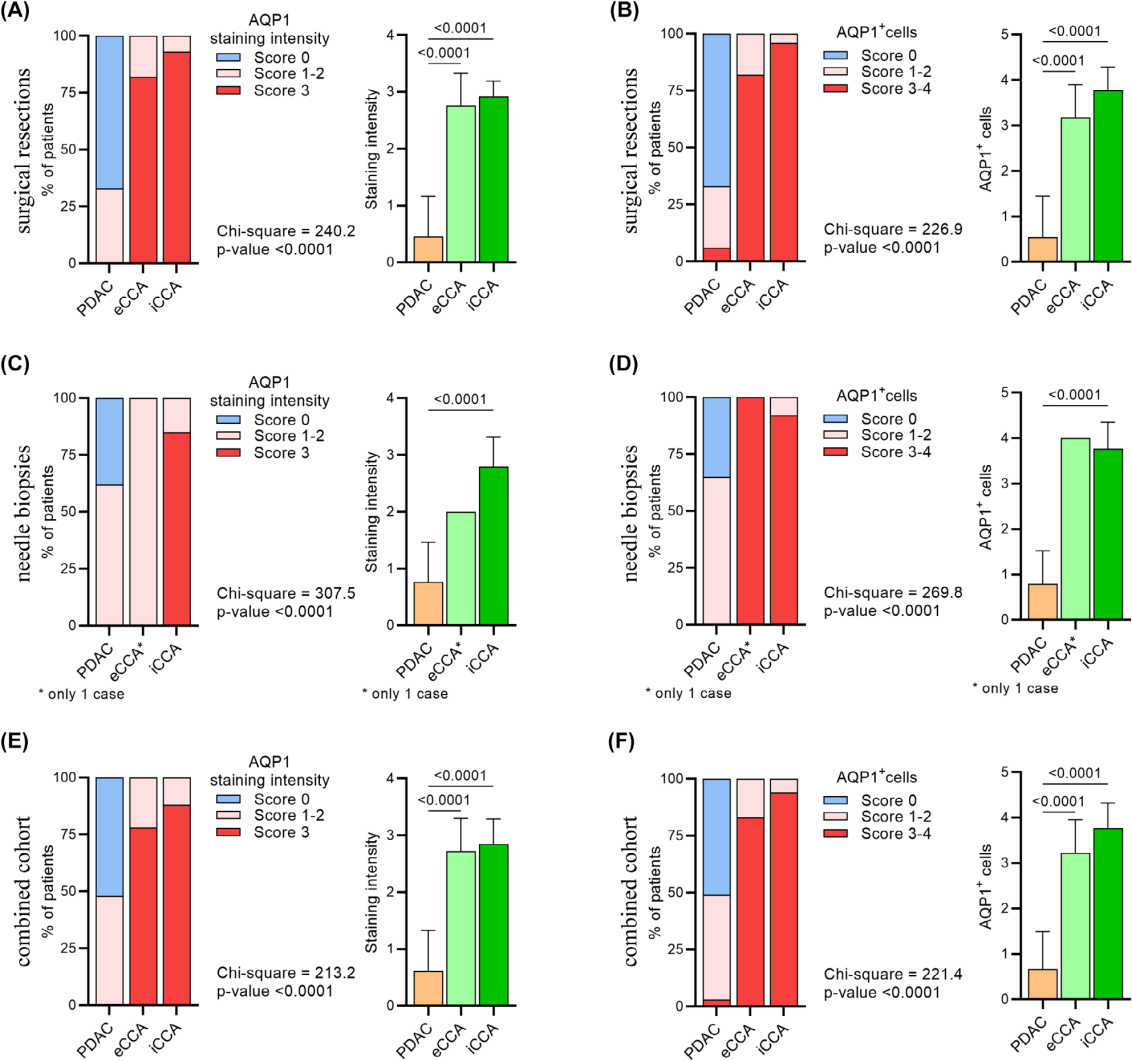
The figure shows the intensity of AQP1 staining (**A**, **C** and **E**) and the percentage of AQP1‐positive neoplastic cells (**B**, **D** and **F**) in CCA (eCCA and iCCA) and PDAC. (**A** and **B**) refer to primary tumours from surgical resections; (**C** and **D**) to needle biopsies; and (**E** and **F**) to the combined analysis of both primary tumours and biopsies. In all panels, the stacked bar graph on the left shows the percentage of patients classified into each score (score 0–3 for AQP1 staining intensity and score 0–4 for the percentage of AQP1‐positive neoplastic cells); while the bar graph on the right shows the mean ± SD of the score for AQP1 staining intensity and the percentage of AQP1‐positive neoplastic cells, respectively. To evaluate statistical differences, the contingency test (chi‐squared test) was used for the stacked bar graphs, while the Kruskal–Wallis test with uncorrected Dunn's post hoc test was used for the bar graphs. [Colour figure can be viewed at wileyonlinelibrary.com]

**Figure 5:**
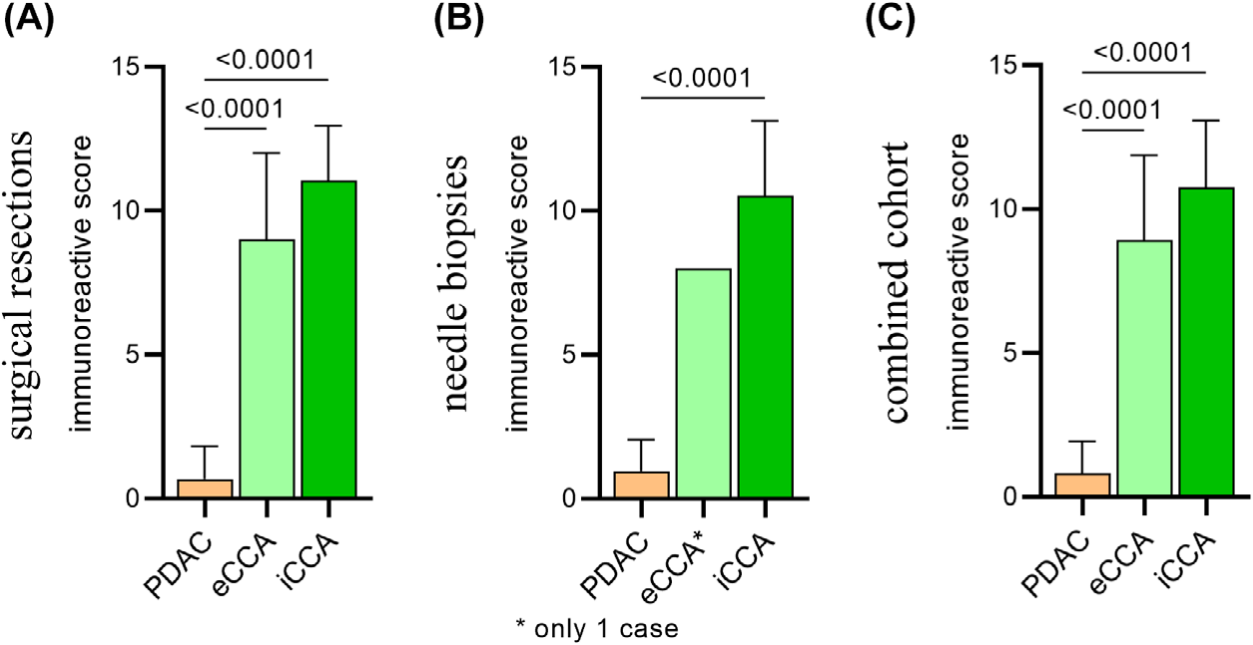
The figure shows the immunoreactive score (IRS) in CCA (eCCA and iCCA) and PDAC in primary tumours from surgical resections (**A**), needle biopsies (**B**) and the combined analysis of both primary tumours and biopsies (**C**). The bar graphs show the mean ± SD of the IRS. To evaluate statistical differences, the Kruskal–Wallis test with uncorrected Dunn's post hoc test was used. [Colour figure can be viewed at wileyonlinelibrary.com]

The comparison between biliary and pancreatic primary tumours showed membranous AQP1 expression in CCA cases, while PDAC cases were negative or weakly positive in a few cells (Figure 3). In particular, AQP1 staining was detected in all 27 iCCAs (mean *staining intensity* score 2.93, with score 1–2 in 7% of cases and 3 in 93%), in all 17 eCCAs (mean *staining intensity* score 2.92, with score 1–2 in 18% of cases and 3 in 82%) and in 11 of 33 PDACs (mean *staining intensity* score 0.45, with score 1–2 in all 11 cases) (Figure 4A). The proportion of AQP1‐positive cells showed a high proportion of AQP1‐positive cells in 26 of 27 iCCAs (mean *proportion of positive cells* score 3.78, with score 1–2 in 4% of cases and 3–4 in 96%) and in 14 of 17 eCCAs (mean *proportion of positive cells* score 3.18, with score 1–2 in 18% and 3–4 in 82%). PDAC stained at a low level in 11 of 33 PDACs (mean *proportion of positive cells* score 0.55, with scores 1–2 in 27% of cases and 3–4 in 6%) (Figure 4B). The mean IRS values of primary tumours from surgical resections were 11.07 for iCCAs (ranging from 6 to 12), 9.00 for eCCAs (ranging from 2 to 12) and 0.7 for PDACs (ranging from 0 to 4) (Figure 5A). The heterogeneity in AQP1 expression was due to the cytoplasmic staining, and only the membranous staining has been considered for IRS.

### AQP1 Expression in CCA Versus mPDAC

The comparison between CCA and mPDAC showed AQP1 staining in all 39 iCCAs (mean *staining intensity* score 2.79, with score 1–2 in 15% of cases and 3 in 85%), in eCCA (*staining intensity* score 2) and in 21 of 34 mPDAC (mean *staining intensity* score 0.76, with score 1–2 in 62% of cases) (Figure 4C). A high proportion of AQP1‐positive cells was detected in 36 of 39 iCCAs (mean *proportion of positive cells* score 3.77, with scores 1–2 in 8% of cases and 3–4 in 92%) and eCCA (*proportion of positive cells* score 4). In iCCA, the immunolabelling was strong, diffuse and membranous, without any significant differences in AQP1 expression between small duct and large duct subtypes. In a smaller proportion, AQP1 was found to be focally expressed in 22 of 34 PDACs (mean *proportion of positive cells* score 0.79, with score 1–2 in 65% of cases) (Figure 4D). The mean IRS values on needle biopsies were 10.54 for iCCAs (ranging from 4 to 12), 8 for the eCCA cases and 0.97 for mPDACs (ranging from 0 to 4) (Figure 5B). The lower AQP1 expression in PDAC was not affected by the histological grade.

### Overall AQP1 Expression in CCA Versus PDAC

The combined analysis of the AQP1 staining in CCA and PDAC highlighted a consistent expression in the two subsets (surgical resections and needle biopsies). AQP1 showed strong membranous expression in iCCA (Figure 4E) (mean *staining intensity* score 2.85), eCCAs stained at slightly lower intensity (mean *staining intensity* score 2.72). Conversely, PDAC displayed either negative or weak AQP1 expression in a few cells (mean *staining intensity* score 0.61). The proportion of AQP1‐positive cells (Figure 4F) was high in iCCAs (mean *proportion of positive cells* score of 3.77) and eCCAs (mean *proportion of positive cells* score of 3.22). Conversely, PDAC showed low expression (mean *proportion of positive cells* score of 0.67), with neither strong nor diffuse reaction in PDAC. The IRS values strengthened these observations, since a high mean IRS value (10.76, range 4–12) was found in iCCA and eCCAs (8.94, range 2–12). PDAC cases showed low mean IRS values (0.84, range 0–4), demonstrating that diffuse and strong AQP1 expression is restricted to CCA (Figure 5C).

### Discriminatory Potential of AQP1

**Figure 6:**
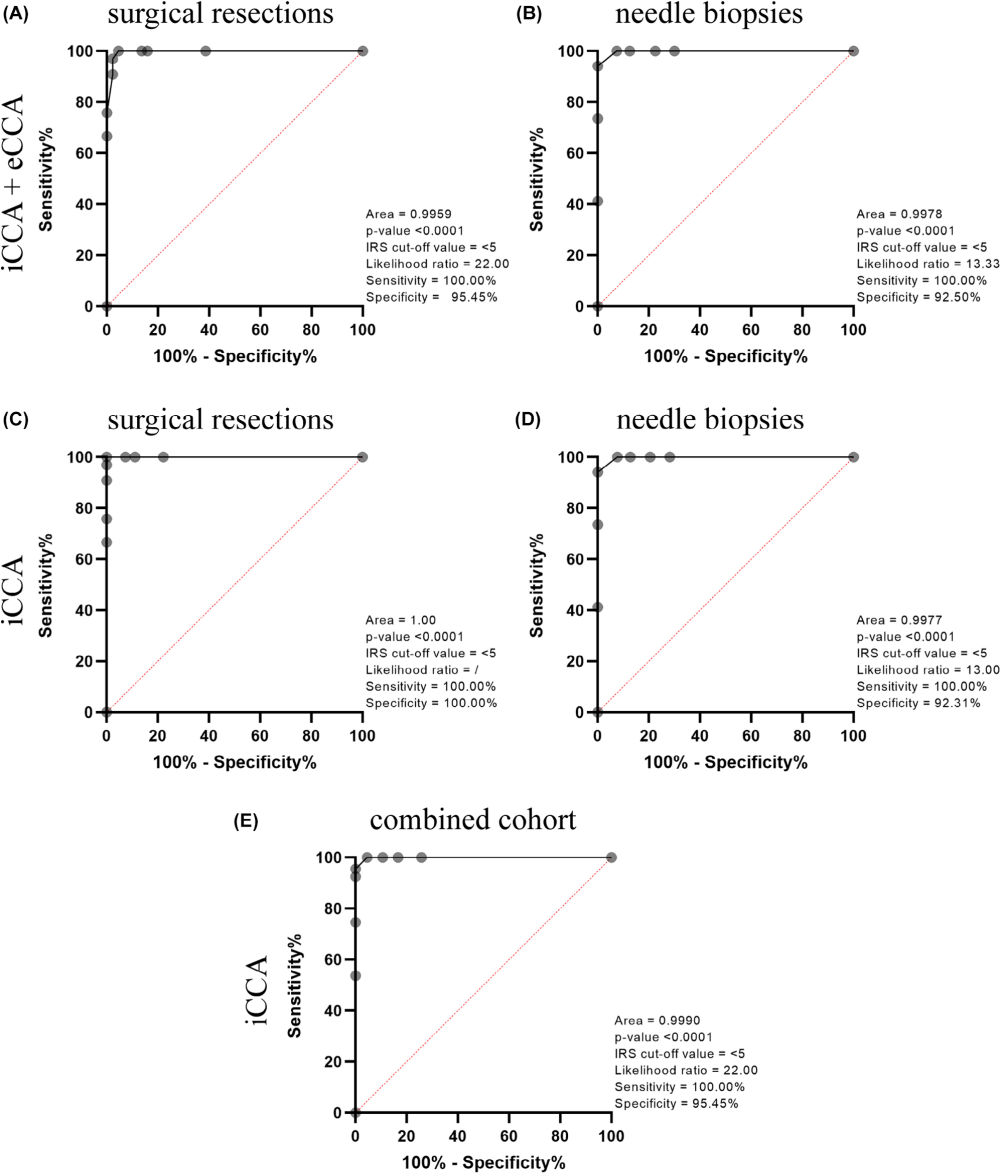
The potential of AQP1 as a diagnostic biomarker for distinguishing CCA from PDAC was assessed using receiver operating characteristic (ROC) curves. This analysis was performed on the combined CCA group (iCCA + eCCA) primary tumours from surgical resections (**A**) and needle biopsies (**B**), in iCCA primary tumours (**C**) and needle biopsies (**D**) and in the combined cohort of both primary tumours and biopsies (**E**). Each ROC curve reports the area under the curve (AUC), the *P*‐value (Wilson/Brown method), the selected IRS cut‐off value, the likelihood ratio and the sensitivity and specificity values. [Colour figure can be viewed at wileyonlinelibrary.com]

The ROC curve analysis confirmed the high discriminatory performance of AQP1 in distinguishing CCA from PDAC. The IRS cut‐off value was selected to ensure 100% sensitivity, maximizing the rate of CCA detection. Comparing primary tumours, AQP1 exhibited good discriminatory power in the CCA versus PDAC comparison. Specifically, the area under the curve (AUC) for CCA (including iCCA and eCCA) was 0.996 in primary tumours, with a specificity of 95.45% (IRS cut‐off value of 5) (Figure 6A); AQP1 showed a similar discriminatory power in iCCA versus mPDAC, with AUC of 0.998 and specificity of 92.50% (IRS cut‐off value of 5) (Figure 6B). Furthermore, AQP1 exhibited an excellent discriminatory ability in identifying iCCA, with an AUC of 1.000 (specificity of 100.00%, IRS cut‐off value of 5) between primary tumours (Figure 6C) and 0.998 (specificity of 92.31%, IRS cut‐off value of 5) between iCCA and mPDAC (Figure 6D), maintaining sensitivity at 100%. Moreover, the combined analysis of both primary tumours and biopsies yielded an AUC of 0.999 and a specificity of 95.45% (IRS cut‐off value of 5), demonstrating an optimal performance in both CCA versus PDAC and iCCA versus mPDAC (Figure 6E). These results highlighted AQP1 as a highly effective diagnostic biomarker in distinguishing iCCA from PDAC.

Focusing on eCCA, the discriminatory power of AQP1 was slightly lower but effective, with an AUC of 0.989 and a specificity of 88.89% (IRS cut‐off value of 5) in the overall analysis (Figure S3). These data suggest that AQP1 confirms its diagnostic performance in eCCA.

Finally, the subanalysis of the ROC curves revealed different predictive power of AQP1 in iCCA exhibiting gland‐forming versus solid histological patterns. It was shown outstanding discrimination for gland‐forming iCCA, with an AUC of 1.000 and specificity of 100% (IRS cut‐off value of 5) (Figure S4A). This result indicates that AQP1 accurately differentiates gland‐forming iCCA from PDAC, with no false‐positive cases observed. In solid iCCA, AQP1 showed an AUC of 0.995 and a specificity of 78.57% (IRS cut‐off value of 5) (Figure S4B). Overall, these data confirmed that AQP1 is an optimal biomarker in discriminating iCCA from mPDAC, particularly in gland‐forming rather than solid iCCA.

## Discussion

Differentiating iCCA from mPDAC poses diagnostic issues.^21^, ^22^ Several biomarkers have been investigated, resulting in little value.^9^, ^23^, ^24^, ^25^ Carbohydrate antigen (CA)19‐9 is routinely used in pancreatobiliary neoplasms, but it is elevated in a broad group of malignancies, including PDAC and CCA.^26^ Serum biomarkers have been investigated,^27^, ^28^ but data are currently inconsistent. The mutational landscape has been proposed as a promising diagnostic tool. KRAS mutations were identified in about 90% of PDAC, as well as other frequent genomic alterations, including TP53, SMAD4 and CDKN2A; CCA was mostly characterized by KRAS, TP53, IDH1/2, ARID1 and FGFR1‐3 fusions. In addition, molecules involved in DNA methylation^29^, ^30^ and multimarker panels^31^, ^32^, ^33^, ^34^ have also been explored. Currently, genomic profiling is increasingly adopted in the diagnostic workflow and can support the distinction between primary and metastatic liver tumours.^35^, ^36^ However, it does not yet provide specific molecular signatures that unequivocally differentiate CCA from PDAC. In this scenario, immunohistochemistry remains a rapid, cost‐effective and widely available tool to improve the diagnostic accuracy of CCA.

AQP1 behaved as a biliary biomarker, accurately discriminating iCCA from mPDAC in liver biopsy. AQP1 exhibited strong membranous expression in bile ducts, benign tumours, pre‐invasive lesions and biliary malignancies. Our comparative evaluation highlighted high sensitivity (100%) and specificity (94.5%) of AQP1, with higher accuracy in gland‐forming iCCA. Both small duct and large duct types were equally stained, without a significant difference (*P*‐value: 0.29) in the expression profile (Figure S5). AQP1 expression was generally absent in PDAC, being weak in a few cancer cells, with the maximum IRS value ≤4. From the diagnostic perspective, it stands for weak‐to‐moderate AQP1 staining in no more than 10% of cancer cells. The IRS cut‐off value was set at <5, which identifies the 10% threshold for histological evaluation. Hence, to overcome the diagnostic dilemma between iCCA and mPDAC raised on liver biopsy specimens, we can state that the evidence of strong membranous AQP1 expression in the most of viable cancer cells (mirroring the IRS value 10%–100% cancer cells) should be considered as suggestive of iCCA; conversely, the absence of AQP1 membranous expression as well as the evidence of weak membranous expression in few cancer cells (mirroring the IRS value 1%–10% cancer cells) should be considered as consistent with mPDAC. In contrast with CCA, which was characterized by homogeneous membranous staining in the vast majority of cancer cells, staining heterogeneity was detected in PDAC, due to the cytoplasmic expression in some cells. If detected, cytoplasmic expression in PDAC was weak and was not considered positive. However, a previous study described AQP1 expression as cytoplasmic in PDAC.^37^ Nevertheless, it should be noted that our investigation focused on the expected location of AQP1 on the cellular membrane. The cited study showed AQP1 expression as cytoplasmic and did not report membranous expression in PDAC. Furthermore, the two studies differ in methodology, primarily concerning the antibody clones used. Our study emphasizes the persistence of AQP1 expression across the entire spectrum of biliary neoplasms, mirroring the expression pattern observed in bile ducts. Accordingly, only the membranous staining pattern was considered suitable for cancer discrimination in the present study. In our series, mPDAC showed a very low level of AQP1, and such results were further validated by imaging findings that confirmed the presence of a pancreatic mass. A small subset of iCCA with solid growth showed lower AQP1 expression as compared to the gland‐forming counterpart. These findings warrant further investigation through immunohistochemical panels, including two or more biomarkers.

In addition, AQP1 can discriminate eCCA from PDAC, as it was also expressed in pCCA and dCCA more than in PDAC, even though at a lower level as compared to iCCA, and representing an intermediate category of staining intensity. Nonetheless, we found that the diagnostic threshold for AQP1 expression can be confirmed for both pCCA and dCCA, since they showed AQP1 membranous expression in more than 10% of cancer cells, ruling out PDAC. Overall, we found that AQP1 can be useful in discriminating eCCA from PDAC, even though with lesser discriminatory potential as a single biomarker, as compared to the diagnostic performance shown in iCCA versus mPDAC differential diagnosis.

**Figure 7:**
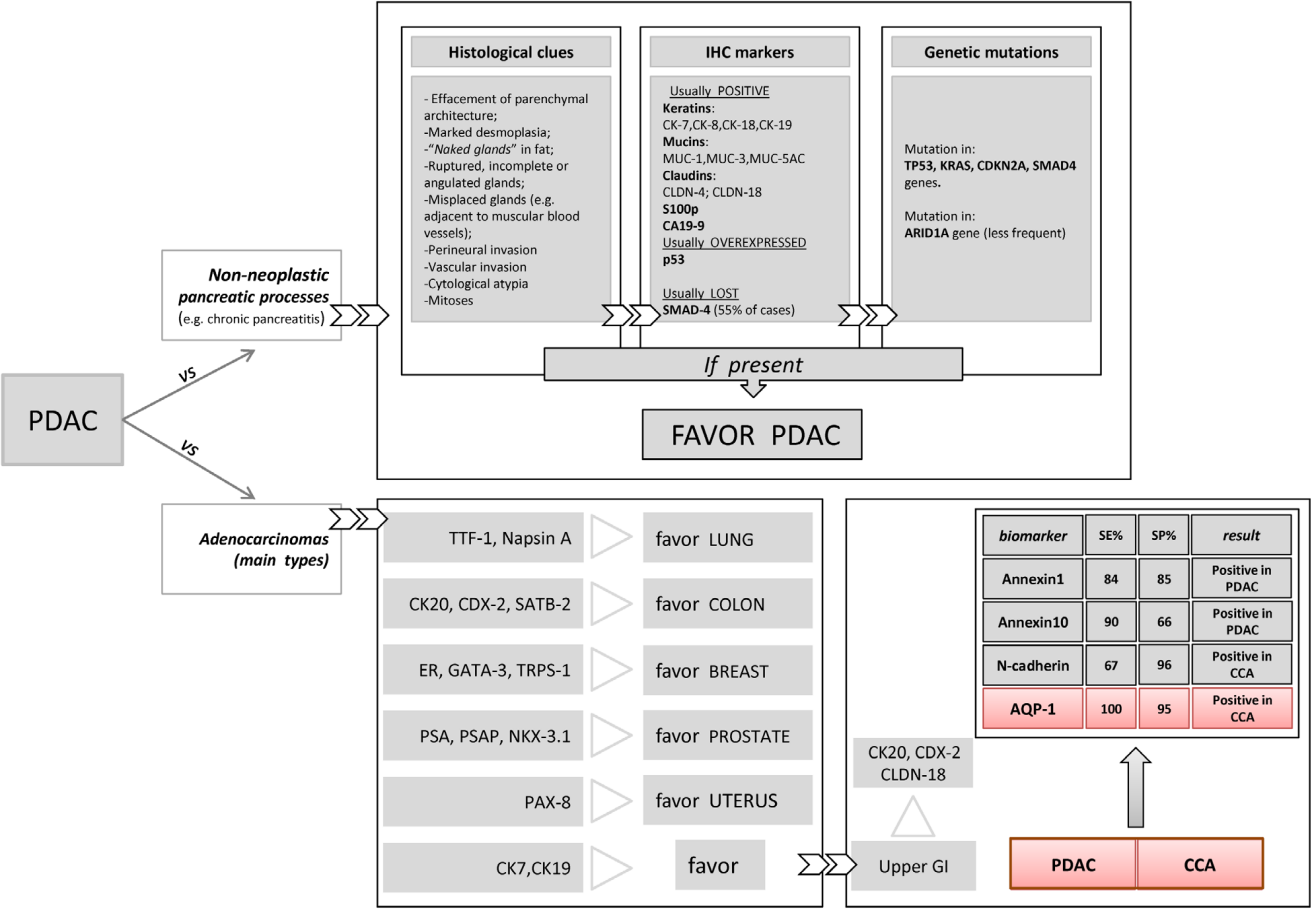
The scheme provides a summary of the PDAC differential diagnosis. The upper panel shows the histological, immunohistochemical and molecular clues for discriminating PDAC from chronic pancreatitis (CP). In the lower panel, the main immunohistochemical antibodies used in the differential diagnosis of adenocarcinoma metastatic to the liver from other primary sites are listed. Note that CCA versus PDAC discrimination is highlighted (*red box*), showing results from several attempts over time to improve the diagnostic accuracy of CCA and PDAC, along with biomarker name, sensitivity and specificity values, and expected results. [Colour figure can be viewed at wileyonlinelibrary.com]

Other putative biomarkers have been recently investigated: Annexins 1 and 10 have been proposed as PDAC biomarkers, whereas N‐cadherin expression has been demonstrated in CCA.^9^, ^25^ As a single biomarker, AQP1 outperformed Annexin 1, Annexin 10 and N‐cadherin, with higher discriminatory power in distinguishing iCCA from mPDAC (Figure 7). Combined immunohistochemical panels including biliary and pancreatic markers still warrant further investigations. Particularly, we could speculate that CA125/MUC16 may represent a candidate biomarker to be evaluated along with AQP1 expression in pancreatobiliary malignancies. CA125/MUC16 has been identified as a downstream target of KRAS, and its expression has been correlated with metabolic reprogramming and aggressiveness in PDAC.^38^, ^39^, ^40^

Furthermore, albumin in situ hybridization (ISH) and genomic abnormalities, including BAP1 loss, have been associated with iCCA. C‐reactive protein (CRP) has been included in a diagnostic panel, being expressed in small duct iCCA.^41^ Caution should be paid, as albumin is a marker of hepatocellular differentiation that has been found in about 80% of iCCA as well as in other extrahepatic malignancies, thus representing a potential diagnostic pitfall.^42^ Furthermore, a comprehensive genomic analysis in a broad iCCA cohort has shown that BAP1 loss accounts for about 15% of cases, and other genomic abnormalities, including IDH1, ARID1, and PRBM1 gene mutations, should be considered in iCCA.^43^ In addition, CRP is not expressed in large duct iCCA as well as in eCCA. Conversely, AQP1 has a broader spectrum of reactivity in biliary tract neoplasms, displaying an optimal discriminatory performance in all the iCCA cases and a good performance in eCCA in our case series. Nonetheless, we found that the comparison between AQP1 and other biliary markers, as well as the experimental validation of the CA125/MUC16 contribution in differentiating iCCA from mPDAC, fell outside the focus of the present paper.

We have focused our line of inquiry on the diagnostic potential of AQP1 as a single biomarker, also providing features on its expression in the liver and pancreatic parenchyma.

Interestingly, membranous AQP1 expression was not found in pancreatic parenchyma, except for the lobular ducts, in line with a previous study.^44^, ^45^ This peculiar AQP1 staining can be applied to routine histology, supporting the differentiation between PDAC and chronic pancreatitis (CP), which is the typical background of PDAC. Although diagnostic criteria have been established, they are not absolute.^46^, ^47^, ^48^ The main diagnostic pitfall in CP is due to parenchymal fibrosis and regressive changes. In our series, CP foci showed small lobular ducts trapped in fibrosis, exhibiting strong AQP1 staining. Conversely, PDAC did not stain, further outlining the discrimination between AQP1‐negative neoplastic glands and AQP1‐positive reactive ducts (Figure 3). Therefore, the role of AQP1 as a diagnostic tool for discriminating PDAC from CP may be hypothesized in selected pancreatic biopsies.

Furthermore, AQP1 expression was found in endothelial cells lining portal vein branches and centrilobular veins, but not liver sinusoids. In experimental models, endothelial AQP1 expression has been detected in vessels close to bile ducts, as seen in human portal tracts, suggesting a functional role in water transport from the bloodstream to bile across cholangiocytes.^49^, ^50^ Such features represent a useful technical control for histological evaluation. In addition, perineurium expressed AQP1, improving in selected cases the detection rate of perineural invasion, a common finding in pancreatobiliary malignancies (Figure S2).

In line with our investigation, transcript analyses confirmed that AQP1 expression is low in PDAC as compared to normal pancreas, suggesting a disruption in AQP1 gene dynamics during carcinogenesis.^51^ AQP1 expression is negatively modulated by miRNA320a, involved in PDAC proliferation, invasion and metastatic spread, suggesting an inverse association between AQP1 expression and PDAC progression.^52^, ^53^

AQP1 expression has been described in other tissues and cancers (Figure S6). Other diagnostic applications have been tested in liver pathology, also including the differentiation between CCA and hepatocellular carcinoma (HCC).^54^ Nevertheless, its diagnostic application as a pan‐biliary biomarker is of paramount relevance, solving the critical dilemma for managing pancreatobiliary malignancies. The expression in benign biliary neoplasms, including biliary adenoma (Figure S7), further supports the idea that the range of AQP1 reactivity in biliary neoplasms is extremely broad. In addition, some authors have evaluated the prognostic role of AQP1. Overall, the prognostic significance of AQP1 in biliary cancers has been described as contradictory: it is associated with aggressiveness and a poor prognosis in eCCA, but with a favourable prognosis in iCCA.^55^, ^56^, ^57^, ^58^

The present study has limitations, mainly due to the lack of comparison with established markers, including albumin ISH, CRP, and BAP1 loss. Furthermore, the study was retrospective and monocentric, investigating a relatively small number of cases.

In conclusion, AQP1 is a highly sensitive and specific biliary biomarker that holds promise in improving the diagnostic accuracy of CCA. As a single immunohistochemical biomarker, AQP1 represents a time‐saving, cost‐effective tool with high yield. Additional diagnostic applications emerged in selected cases.

## Author contributions

LG: Study design and conceptualization, draft writing and critical revision; BM: draft conceptualization and data collection and analysis; DBM: draft writing and critical revision; CL: statistical analyses and draft writing; BB: data and images collection; GM: data and images collection; RS: draft writing and critical revision; MV: study design and critical revision.

## Funding information

The present study received no specific funds.

## Conflict of interest statement

None to declare.

## Supporting information


**Figure S1.** The variability in AQP1 staining in pancreatobiliary malignancies.

**Figure S2.** AQP1 expression in non‐biliary structures.

**Figure S3.** Potential of AQP1 as a diagnostic biomarker for distinguishing eCCA from PDAC.

**Figure S4.** Potential of AQP1 as a diagnostic biomarker for distinguishing iCCA with different histological pattern from PDAC.

**Figure S5.** Comparison of the immunoreactive score (IRS) in the small duct and large duct subtypes of iCCA.

**Figure S6.** AQP1 RNA expression in tumours and corresponding normal tissues from the TGCA database.

**Figure S7.** Biliary adenoma.

**Table S1.** Clinical–pathological data of samples from surgical resections.

**Table S2.** Clinical–pathological data of samples from needle biopsies.

## Data Availability

The data that support the findings of this study are available from the corresponding author upon reasonable request.
