# Supplementary figures and images for: Aquaporin‐1 differentiates intrahepatic cholangiocarcinoma from liver metastases of pancreatic ductal adenocarcinoma
Source: Histopathology. 2026 Jan 29;88(7):1360–72. doi: 10.1111/his.70108 (PMC13128327; doi:10.1111/his.70108)

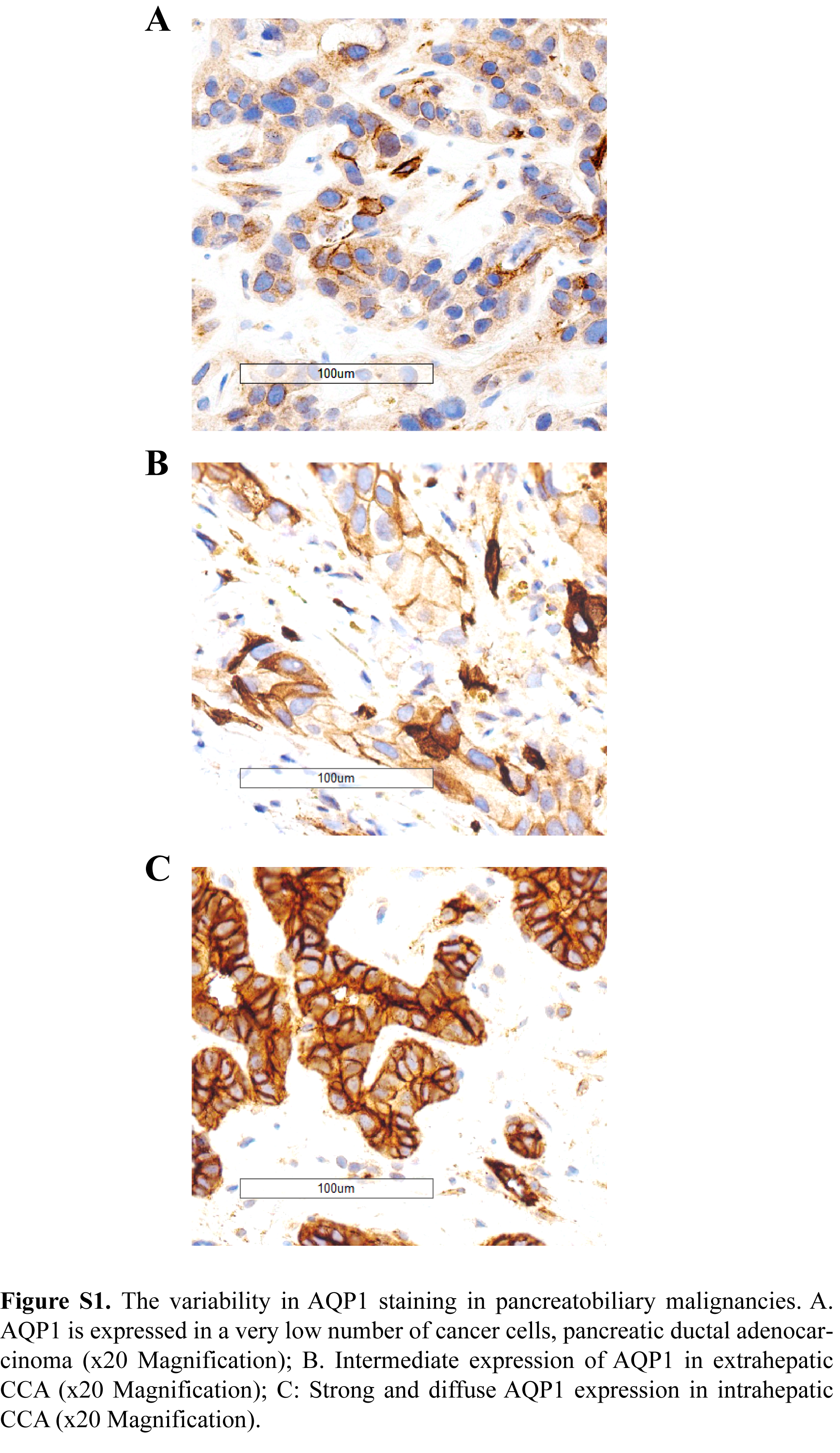

Supplement: Supplementary file 1 — Figure S1. The variability in AQP1 staining in pancreatobiliary malignancies. [file HIS-88-1360-s008.tif]

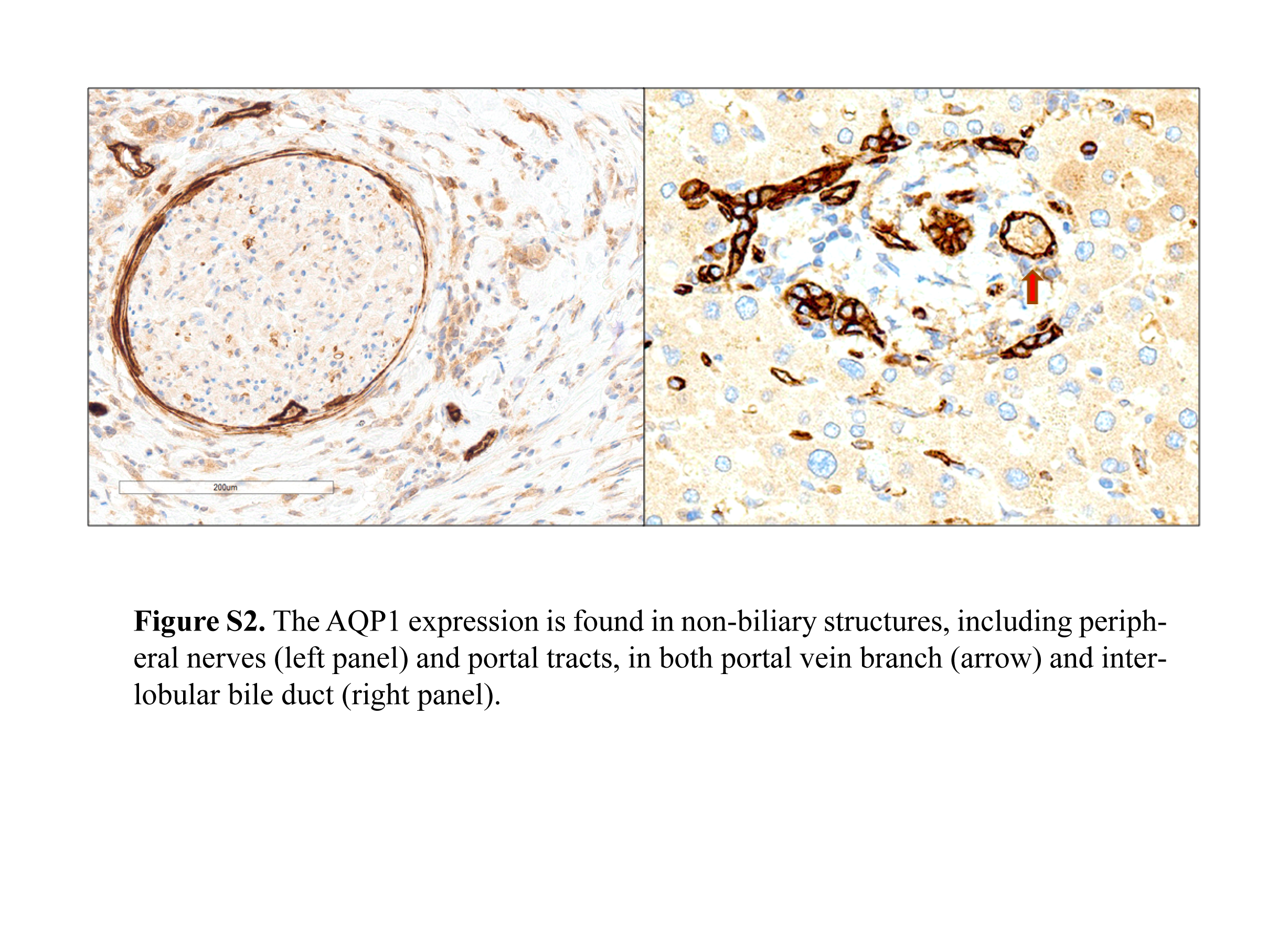

Supplement: Supplementary file 2 — Figure S2. AQP1 expression in non‐biliary structures. [file HIS-88-1360-s006.tif]

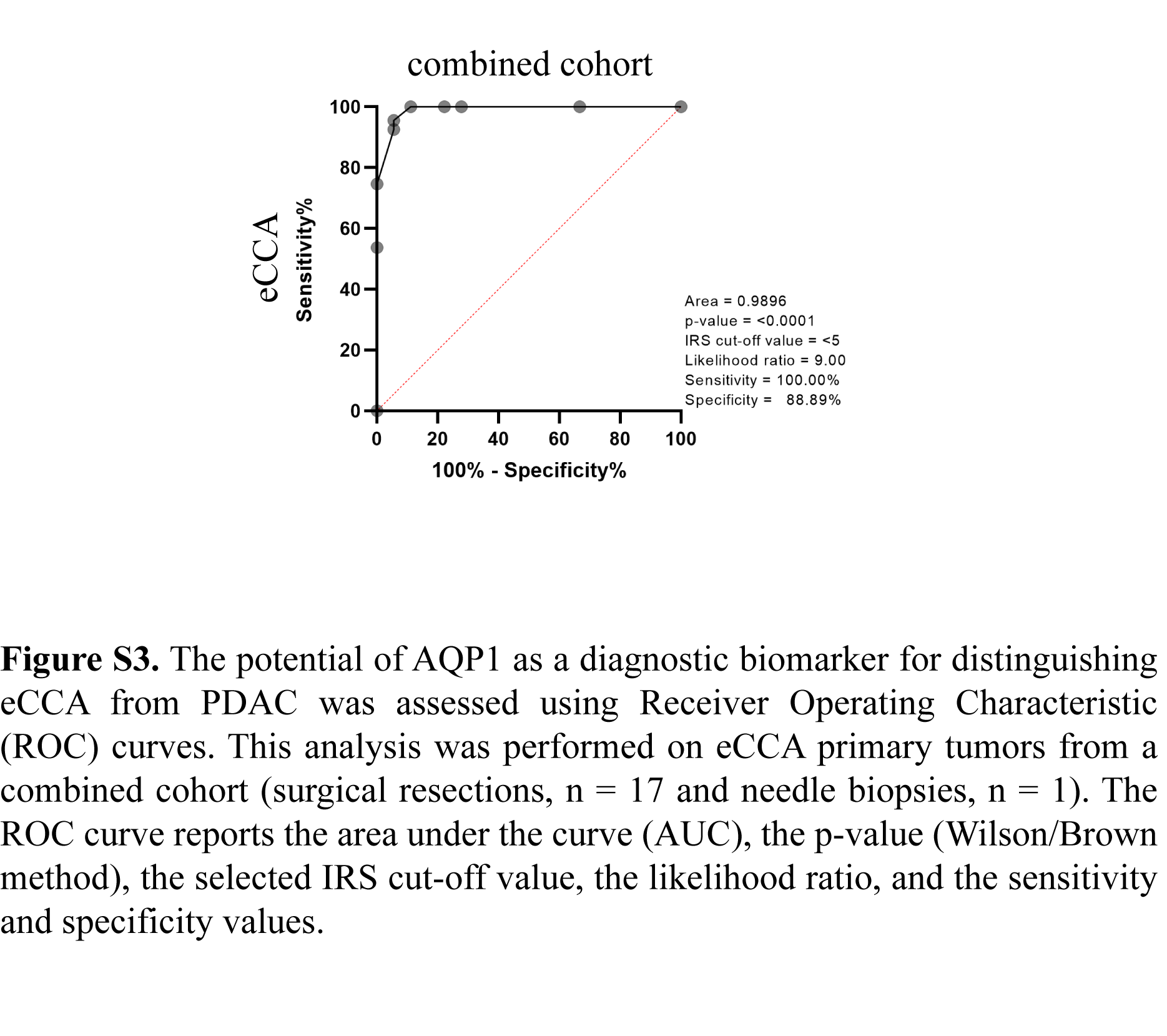

Supplement: Supplementary file 3 — Figure S3. Potential of AQP1 as a diagnostic biomarker for distinguishing eCCA from PDAC. [file HIS-88-1360-s009.tif]

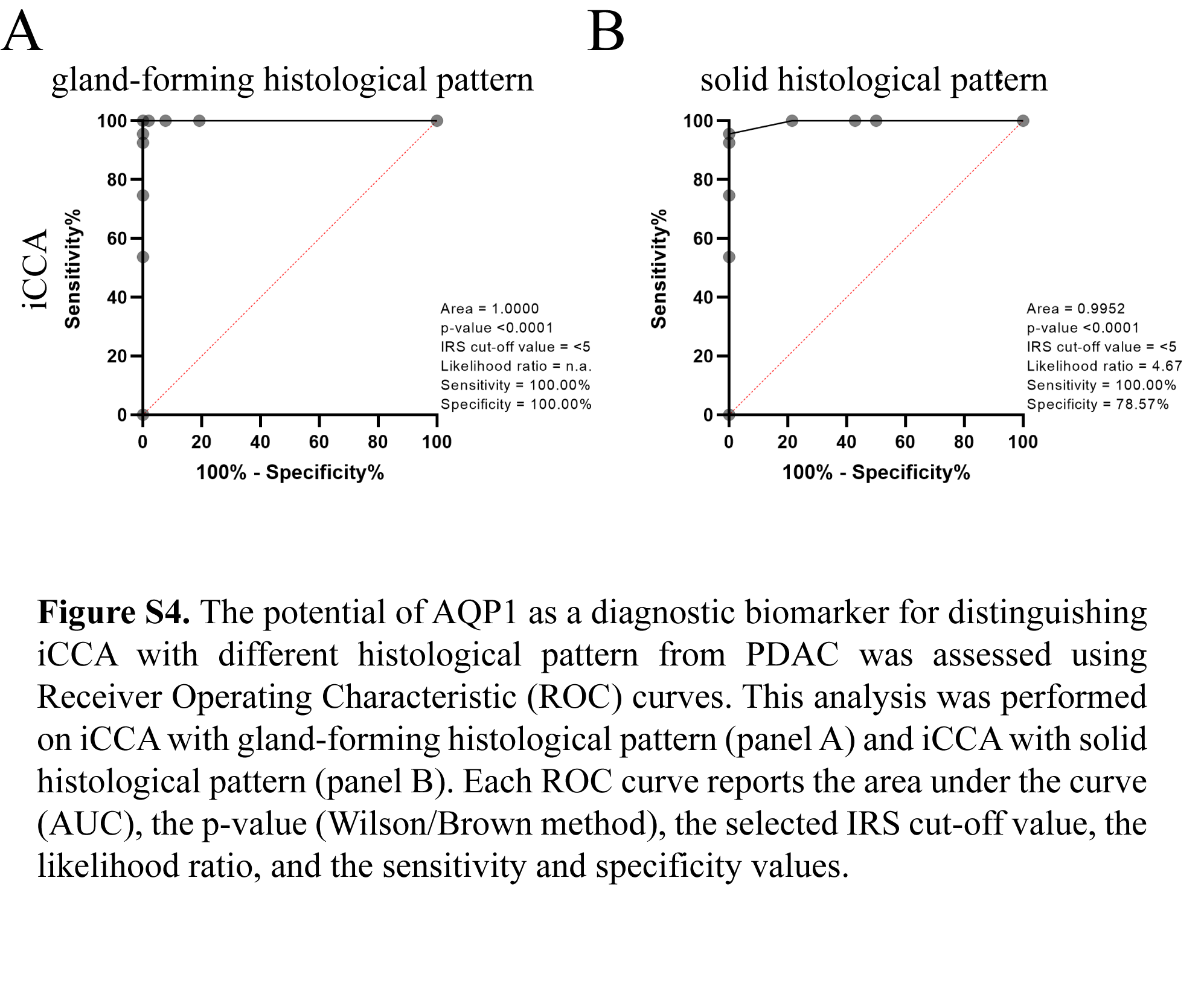

Supplement: Supplementary file 4 — Figure S4. Potential of AQP1 as a diagnostic biomarker for distinguishing iCCA with different histological pattern from PDAC. [file HIS-88-1360-s003.tif]

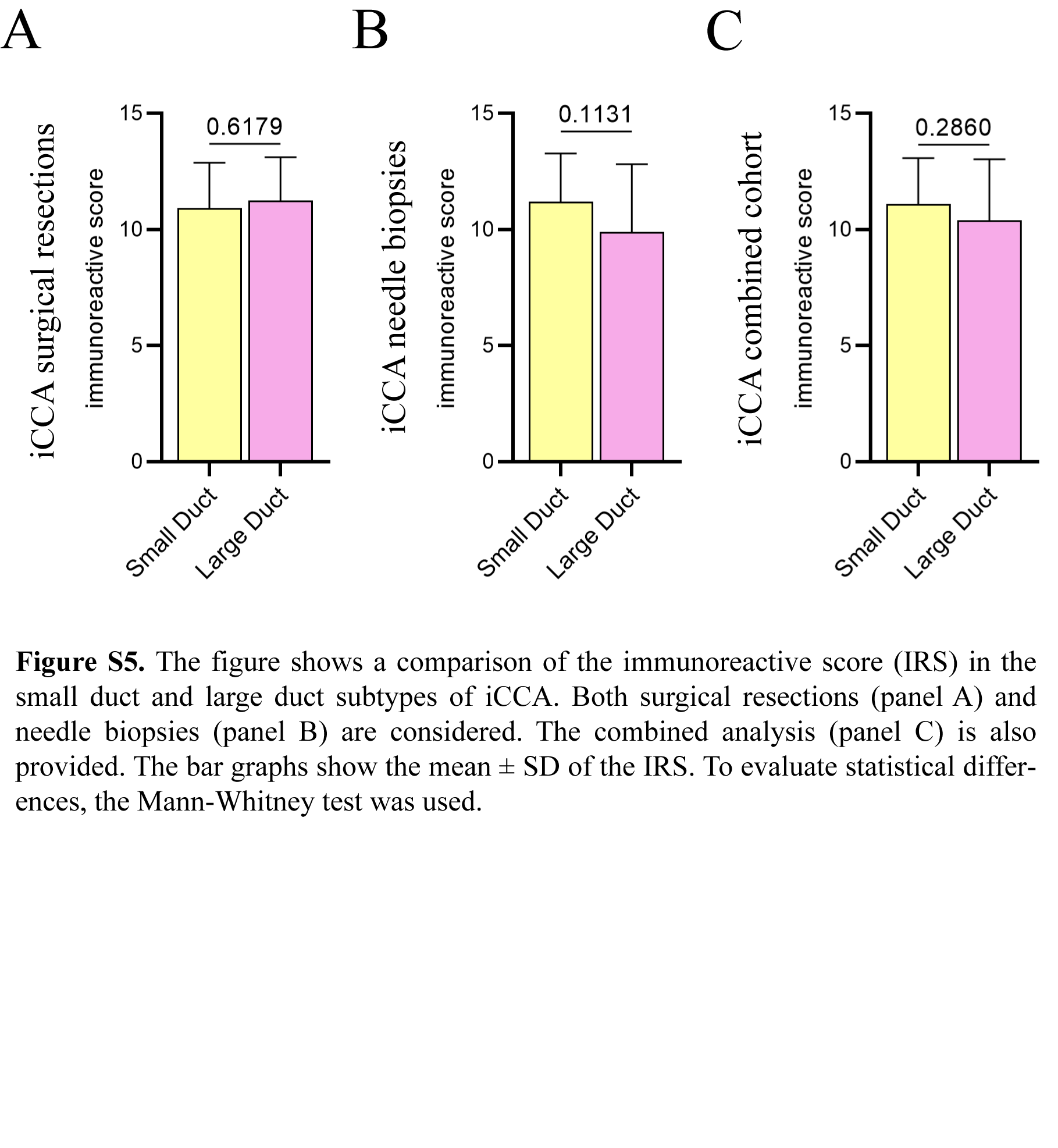

Supplement: Supplementary file 5 — Figure S5. Comparison of the immunoreactive score (IRS) in the small duct and large duct subtypes of iCCA. [file HIS-88-1360-s005.tif]

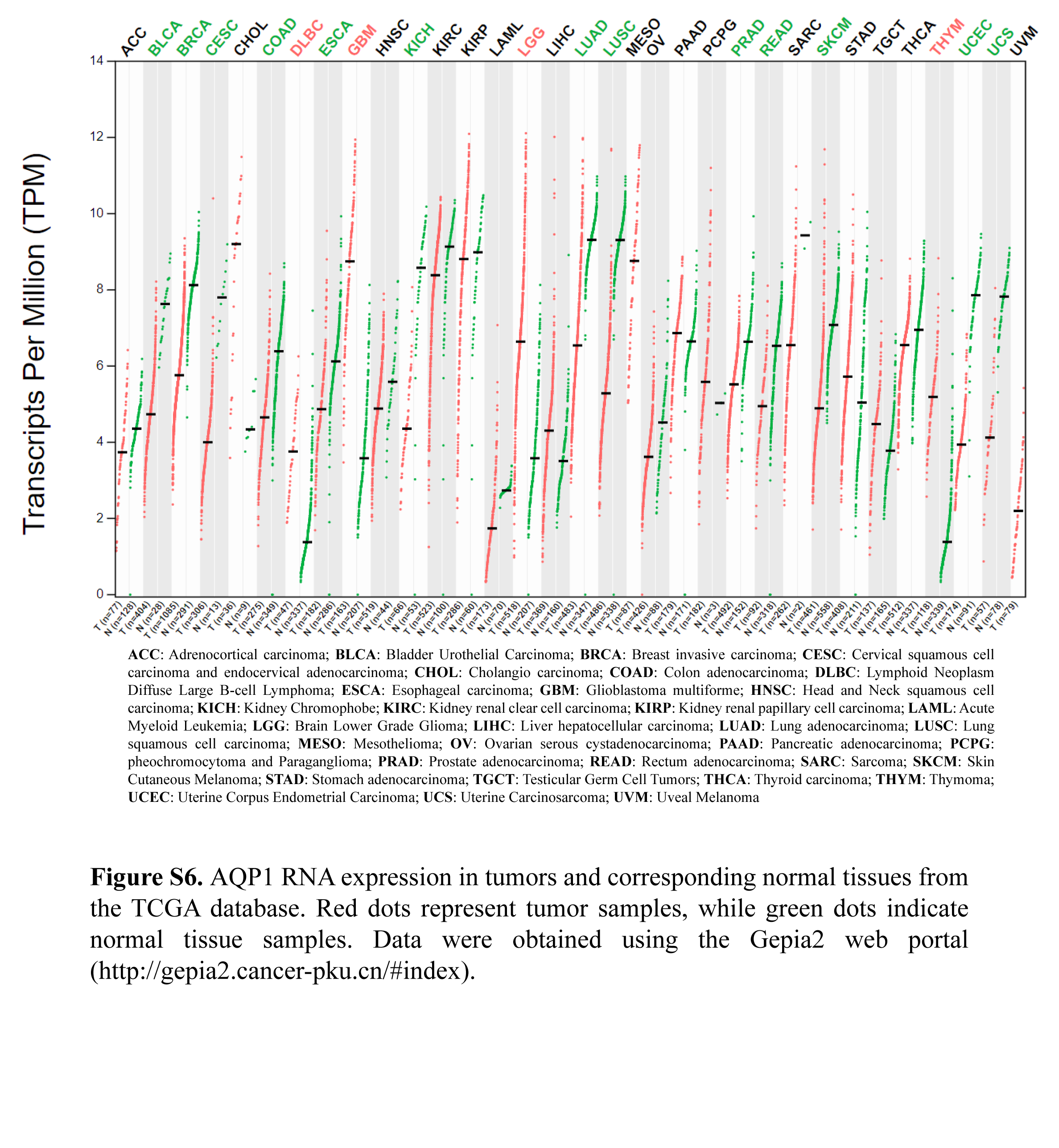

Supplement: Supplementary file 6 — Figure S6. AQP1 RNA expression in tumours and corresponding normal tissues from the TGCA database. [file HIS-88-1360-s007.tif]

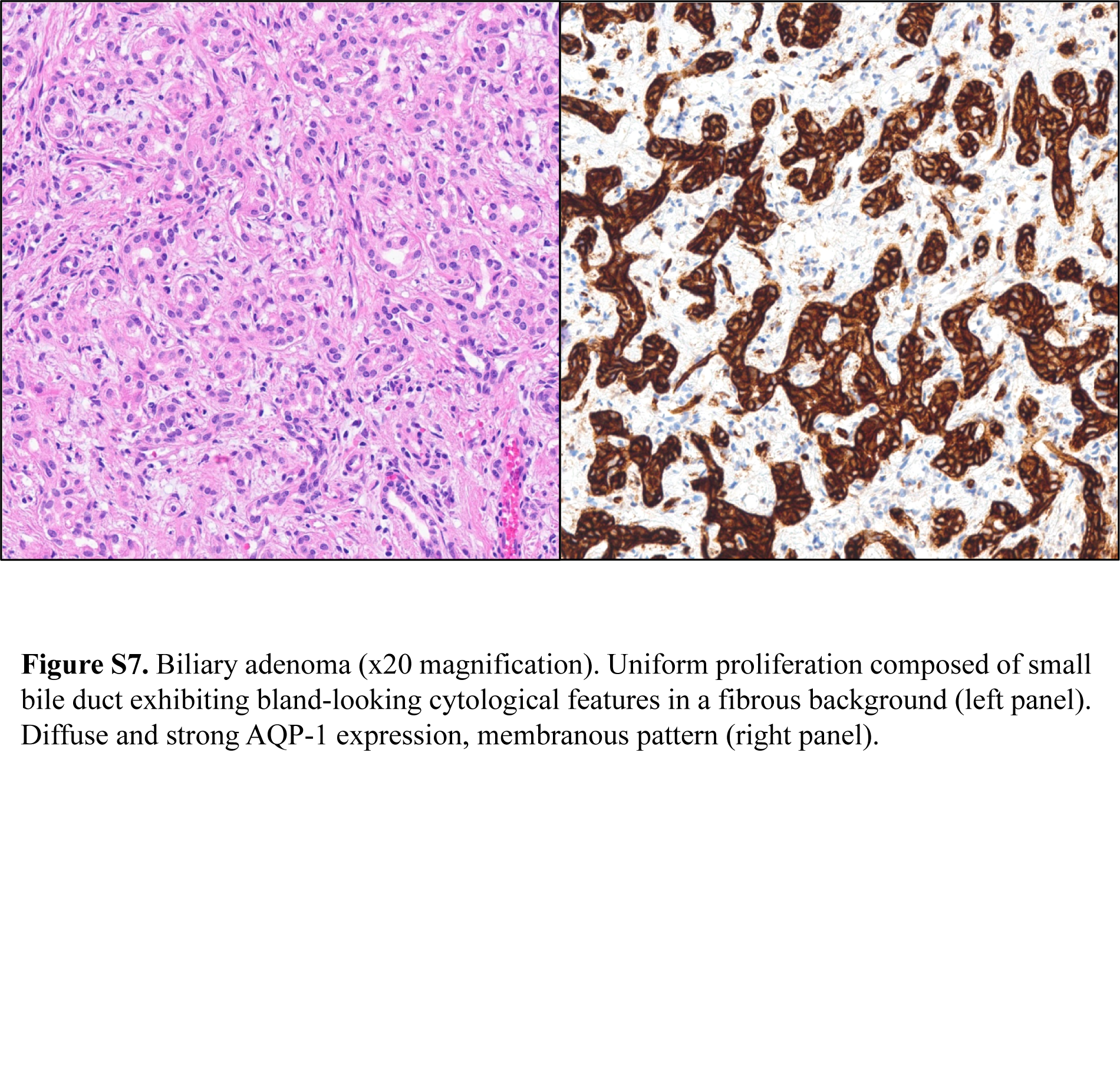

Supplement: Supplementary file 7 — Figure S7. Biliary adenoma. [file HIS-88-1360-s001.tif]
