# Supplementary material for: Aquaporin‐1 differentiates intrahepatic cholangiocarcinoma from liver metastases of pancreatic ductal adenocarcinoma
Source: Histopathology. 2026 Jan 29;88(7):1360–72. doi: 10.1111/his.70108 (PMC13128327; doi:10.1111/his.70108)
Supplement: Supplementary file 8 — Table S1. Clinical–pathological data of samples from surgical resections. [file HIS-88-1360-s002.pdf]

Table S1. Clinical-pathological data of samples from surgical resections

Case n°	Sex/Age	Procedure	Histology	iCCA Subtype	T-Grade	p-TNM	c-Stage	Pattern	AQP1		
									**proportion of positive cells**<br>0: no positive cells<br>1: 1-5% of cells<br>2:6-10% of cells<br>3: 11-50% of cells<br>4: 51-100% of cells	**staining intensity**<br>0: none<br>1: weak<br>2: moderate<br>3: strong	**immunoreactive score (IRS)**
SRe1	F55	DCP	e-CCA (d-CCA)	n.a.	G2	T2,N0,M0	II A	Gland-forming	3	2	6
SRe2	M68	DCP	e-CCA (d-CCA)	n.a.	G2	T1,N0,M0	I	Gland-forming	3	3	9
SRe3	F78	DCP	e-CCA (d-CCA)	n.a.	G2	T3, N1,M0	II B	Gland-forming	4	3	12
SRe4	M41	DCP	e-CCA (d-CCA)	n.a.	G2	y,T2,N0,M0	II A	Gland-forming	3	3	9
SRe5	M57	DCP	e-CCA (d-CCA)	n.a.	G2	T2,N0,M0	II A	Gland-forming	4	3	12
SRe6	F61	DCP	e-CCA (d-CCA)	n.a.	G2	T3,N2,M0	III A	Gland-forming	3	3	9
SRe7	M72	PLR	e-CCA (d-CCA)	n.a.	G2	T2a,N1	III B	Gland-forming	3	3	9
SRe8	M77	WCh	e-CCA (d-CCA)	n.a.	G2	T1,N1,M0	II A	Gland-forming	3	3	9
SRe9	F/81	WCh	e-CCA (d-CCA)	n.a.	g1	T1b;N0	I B	Gland-forming	4	3	12
SRe10	M86	WCh	e-CCA (d-CCA)	n.a.	G2	T3,N1,M0	III B	Gland-forming	4	3	12
SRe11	F66	WCh	e-CCA (d-CCA)	n.a.	G2	T3,N2,M0	III A	Gland-forming	2	3	6
SRe12	F55	Wch	e-CCA (d-CCA)	n.a.	G3	T2a,N0,M0	II A	Gland-forming	4	3	12
SRe13	F57	LT	e-CCA (p-CCA)	n.a.	G3	T2b,N1,M1	IV B	Gland-forming	2	2	4
SRe14	M54	LT	e-CCA (p-CCA)	n.a.	G2	T3,N2,M1	IV B	Gland-forming	2	1	2
SRe15	F62	LT	e-CCA (p-CCA)	n.a.	G3	T2b,N1,M1	III C	Gland-forming	4	3	12
SRe16	F68	LT	e-CCA (p-CCA)	n.a.	G2	T3,N1,M0	III C	Gland-forming	3	3	9
SRe17	M62	LT	e-CCA (p-CCA)	n.a.	G2	yT1, N0,M0	I	Gland-forming	3	3	9
SRi1	M58	LT	i-CCA	Small duct	G3	yT2,N0,M0	II	Solid	4	3	12
SRi2	M59	LT	i-CCA	Small duct	G3	yT2,N0,M0	II	Gland-forming	4	3	12
SRi3	F70	LT	i-CCA	Large duct	G2	y,T1a,N0,M0	I A	Gland-forming	4	3	12
SRi4	F84	PLR	i-CCA	Large duct	G3	T2,N0,M0	II	Solid	2	3	6
SRi5	F46	PLR	i-CCA	Small duct	G3	T1,N0,M0	I	Gland-forming	4	2	8
SRi6	F80	PLR	i-CCA	Large duct	G3	T1,N0,M0	I	Gland-forming	4	3	12
SRi7	F72	PLR	i-CCA	Small duct	G1	T1a,N0,M0	I	Gland-forming	4	3	12
SRi8	F69	PLR	i-CCA	Small duct	G2	T2, N1,M0	III B	Gland-forming	4	3	12
SRi9	M72	PLR	i-CCA	Large duct	G3	T2, N1,M1	IV	Gland-forming	4	3	12
SRi10	F52	PLR	i-CCA	Large duct	G3	T2,N0,M0	II	Gland-forming	4	3	12
SRi11	F74	PLR	i-CCA	Large duct	G3	T2,N0,M0	II	Gland-forming	4	3	12
SRi12	F79	PLR	i-CCA	Small duct	G3	T2,N0,M0	II	Solid	3	2	6
SRi13	F79	PLR	i-CCA	Large duct	G3	T2,N0,M0	II	Gland-forming	4	3	12
SRi14	M48	PLR	i-CCA	Small duct	G1	T2,N0,M0	II	Gland-forming	4	3	12
SRi15	M49	PLR	i-CCA	Large duct	G3	T2,N0,M0	II	Gland-forming	4	3	12
SRi16	M52	PLR	i-CCA	Large duct	G3	T2,N0,M0	II	Gland-forming	4	3	12
SRi17	M73	PLR	i-CCA	Small duct	G3	T2,N0,M0	II	Solid	4	3	12
SRi18	M76	PLR	i-CCA	Small duct	G2	T2,N0,M0	II	Gland-forming	4	3	12
SRi19	M76	PLR	i-CCA	Small duct	G3	T2,N1,M0	III B	Gland-forming	3	3	9
SRi20	M61	PLR	i-CCA	Small duct	G3	T2;N0;M1	IV	Gland-forming	4	3	12
SRi21	M63	PLR	i-CCA	Small duct	G3	T4,N0,M0	III B	Solid	4	3	12
SRi22	M66	PLR	i-CCA	Small duct	G2	yT1, N0,M0	I	Gland-forming	3	3	9
SRi23	M68	PLR	i-CCA	Large duct	G2	yT2,N0,M0	II	Gland-forming	4	3	12
SRi24	M72	PLR	i-CCA	Large duct	G3	yT2,N0,M0	II	Gland-forming	4	3	12
SRi25	F69	PLR	i-CCA	Small duct	G2	T2,N1,M0	IIII B	Gland-forming	4	3	12
SRi26	F66	PLR	i-CCA	Large duct	G2	T1a,N0,M0	I A	Gland-forming	3	3	9
SRi27	F74	PLR	i-CCA	Small duct	G2	T1b, N0,M0	I B	Gland-forming	4	3	12
SRp1	M58	DCP	PDAC	n.a.	G2	T2,N2,M1	IV	Gland-forming	1	1	1
SRp2	F68	DCP	PDAC	n.a.	G3	T3,N1,M0	III A	Gland-forming	0	0	0
SRp3	F70	DCP	PDAC	n.a.	G2	T3b;N2;M0	III B	Gland-forming	1	1	1
SRp4	M54	DCP	PDAC	n.a.	G3	T3b;N2;M0	III B	Gland-forming	0	0	0
SRp5	F63	DCP	PDAC	n.a.	G3	T1c,N1,M0	II B	Solid	0	0	0
SRp6	F79	DCP	PDAC	n.a.	G2	T2,N1,M0	II B	Gland-forming	2	1	2
SRp7	F57	DCP	PDAC	n.a.	G2	T2,N2,M0	III	Gland-forming	0	0	0
SRp8	F71	DCP	PDAC	n.a.	G3	T2,N2,M0	III	Solid	0	0	0
SRp9	F72	DCP	PDAC	n.a.	G2	T2,N2,M0	III	Gland-forming	0	0	0
SRp10	M57	DCP	PDAC	n.a.	G1	T2,N2,M0	III	Gland-forming	0	0	0
SRp11	M64	DCP	PDAC	n.a.	G3	T2,N2,M0	III	Gland-forming	0	0	0
SRp12	F53	DCP	PDAC	n.a.	G3	T3,N1,M0	II B	Gland-forming	2	2	4
SRp13	M84	DCP	PDAC	n.a.	G3	T3,N1,M0	II B	Gland-forming	0	0	0
SRp14	F74	DCP	PDAC	n.a.	G2	T3,N2,M0	III	Gland-forming	2	1	2
SRp15	F/50	DCP	PDAC	n.a.	G2	T2;N2;M1	IV	Gland-forming	0	0	0
SRp16	F/77	DCP	PDAC	n.a.	G3	T2;N1	iII B	Gland-forming	1	1	1
SRp17	F76	DCP	PDAC	n.a.	G2	yT2,N2,M1	IV	Gland-forming	0	0	0
SRp18	F75	DCP	PDAC	n.a.	G3	yT1,N1,M0	II B	Gland-forming	0	0	0
SRp19	F74	DCP	PDAC	n.a.	G2	T2,N0,M0	II A	Gland-forming	3	1	3
SRp20	M62	DCP	PDAC	n.a.	G3	T2,N2,M1	IV	Gland-forming	1	2	2
SRp21	M66	DCP	PDAC	n.a.	G2	y,T2,N0,M0	I B	Gland-forming	1	2	2
SRp22	F67	DCP	PDAC	n.a.	G2	y,T2,N1,M0	II B	Gland-forming	3	1	3
SRp23	M63	DCP	PDAC	n.a.	G2	pT2,N2,M0	III	Gland-forming	0	0	0
SRp24	M71	DCP	PDAC	n.a.	G2	pT2,N2,M0	III	Gland-forming	0	0	0
SRp25	M79	DCP	PDAC	n.a.	G2	y,T2,N0,M0	I B	Gland-forming	0	0	0
SRp26	F85	DCP	PDAC	n.a.	G2	T3,N0,M0	II A	Gland-forming	0	0	0
SRp27	F64	DCP	PDAC	n.a.	G2	T2,N20,M0	I B	Gland-forming	0	0	0
SRp28	M81	DCP	PDAC	n.a.	G2	T3b,N0,M0	II B	Gland-forming	1	2	2
SRp29	F77	DCP	PDAC	n.a.	G2	T3b,N2,M0	III B	Gland-forming	0	0	0
SRp30	M52	DCP	PDAC	n.a.	G2	T3b,N1,M0	III A	Gland-forming	0	0	0
SRp31	F80	DCP	PDAC	n.a.	G2	T3b,N1,M0	III A	Gland-forming	0	0	0
SRp32	F63	DPR	PDAC	n.a.	G2	T3,N0,M1	IV	Gland-forming	0	0	0
SRp33	M84	DPR	PDAC	n.a.	G2	T3,N2,M0	III B	Gland-forming	0	0	0

DCP *: duodenocefalopancreasectomy;* ***LT*** *: liver transplantation;* ***PLR*** *: partial liver resection;* ***WCh:*** *Wide cholecystectomy;* ***DPR:*** *distal pancreatic resection* ***PDAC*** *: pancreatic ductal adenocarcinoma* ***CCA*** *: cholangiocarcinoma*
n.a. *not assessed*
