# Supplementary material for: Aquaporin‐1 differentiates intrahepatic cholangiocarcinoma from liver metastases of pancreatic ductal adenocarcinoma
Source: Histopathology. 2026 Jan 29;88(7):1360–72. doi: 10.1111/his.70108 (PMC13128327; doi:10.1111/his.70108)
Supplement: Supplementary file 9 — Table S2. Clinical–pathological data of samples from needle biopsies. [file HIS-88-1360-s004.pdf]

Table S2. Clinical-pathological data of samples from needle biopsies

							AQP1		
**Case n°**	**Sex/Age**	**Primary Tumor**	**Histology**	**iCCA Subtype**	**T-Grade**	**Pattern**	**proportion of positive cells**<br>0: no positive cells<br>1: 1-5% of cells<br>2:6-10% of cells<br>3: 11-50% of cells<br>4: 51-100% of cells	**staining intensity**<br>0: none<br>1: weak<br>2: moderate<br>3: strong	**immunoreactive score (IRS)**
LBe1	F67	Choled.	e-CCA (d-CCA)	n.a.	G3	n.a.	4	2	8
LBi1	M63	Liver	iCCA	Large duct	G3	Solid	4	1	4
LBi2	M65	Liver	iCCA	Large duct	G3	Solid	2	2	4
LBi3	F/80	Liver	iCCA	Small duct	G3	Solid	4	2	8
LBi4	M/64	Liver	iCCA	Large duct	G3	Solid	2	3	6
LBi5	M29	Liver	iCCA	Small duct	G3	Solid	4	1	4
LBi6	M60	Liver	iCCA	Small duct	G3	Solid	4	3	12
LBi7	M75	Liver	iCCA	Large duct	G3	Solid	4	3	12
LBi8	M53	Liver	iCCA	Small duct	G2	Gland-forming	4	3	12
LBi9	F58	Liver	iCCA	Large duct	G2	Gland-forming	4	3	12
LBi10	M55	Liver	iCCA	Small duct	G2	Gland-forming	4	3	12
LBi11	F57	Liver	iCCA	Large duct	G2	Gland-forming	4	3	12
LBi12	M80	Liver	iCCA	Large duct	G2	Gland-forming	4	3	12
LBi13	F58	Liver	iCCA	Large duct	G2	Gland-forming	4	3	12
LBi14	F64	Liver	iCCA	Large duct	G2	Gland-forming	4	3	12
LBi15	F74	Liver	iCCA	Small duct	G2	Gland-forming	4	3	12
LBi16	M/56	Liver	iCCA	Large duct	G3	Gland-forming	3	3	9
LBi17	M/66	Liver	iCCA	Large duct	G3	Gland-forming	2	3	6
LBi18	M/56	Liver	iCCA	Large duct	G2	Gland-forming	4	2	8
LBi19	F/60	Liver	iCCA	Small duct	G3	Gland-forming	4	3	12
LBi20	M/67	Liver	iCCA	Small duct	G3	Gland-forming	4	3	12
LBi21	F/64	Liver	iCCA	Large duct	G2	Gland-forming	3	3	9
LBi22	F47	Liver	iCCA	Large duct	G2	Gland-forming	4	3	12
LBi23	F59	Liver	iCCA	Small duct	G2	Gland-forming	4	3	12
LBi24	M66	Liver	iCCA	Small duct	G3	Gland-forming	3	3	9
LBi25	M65	Liver	iCCA	Large duct	G2	Gland-forming	4	3	12
LBi26	M84	Liver	iCCA	Small duct	G2	Gland-forming	4	3	12
LBi27	M72	Liver	iCCA	Small duct	G3	Gland-forming	4	3	12
LBi28	M57	Liver	iCCA	Large duct	G2	Gland-forming	4	3	12
LBi29	M63	Liver	iCCA	Small duct	G2	Gland-forming	4	3	12
LBi30	F70	Liver	iCCA	Small duct	G3	Solid	4	3	12
LBi31	F71	Liver	iCCA	Large duct	G2	Gland-forming	4	2	8
LBi32	F65	Liver	iCCA	Small duct	G3	Gland-forming	4	3	12
LBi33	F77	Liver	iCCA	Small duct	G3	Solid	4	3	12
LBi34	M54	Liver	iCCA	Small duct	G2	Gland-forming	4	3	12
LBi35	M49	Liver	iCCA	Small duct	G2	Gland-forming	4	3	12
LBi36	M71	Liver	iCCA	Small duct	G3	Gland-forming	4	3	12
LBi37	M71	Liver	iCCA	Large duct	G2	Gland-forming	4	3	12
LBi38	F65	Liver	iCCA	Large duct	G3	Gland-forming	4	3	12
LBi39	M73	Liver	iCCA	Large duct	G2	Gland-forming	4	3	12
LBp1	M70	Ampulla	PDAC	n.a.	G3	n.a.	1	1	1
LBp2	F68	Ampulla	PDAC	n.a.	G3	n.a.	1	1	1
LBp3	F82	Pancreas	PDAC	n.a.	G3	n.a.	1	2	2
LBp4	M64	Pancreas	PDAC	n.a.	G3	n.a.	1	1	1
LBp5	F76	Pancreas	PDAC	n.a.	G3	n.a.	2	1	2
LBp6	F82	Pancreas	PDAC	n.a.	G2	n.a.	1	2	2
LBp7	F62	Pancreas	PDAC	n.a.	G2	n.a.	0	0	0
LBp8	F69	Pancreas	PDAC	n.a.	G3	n.a.	0	0	0
LBp9	M78	Pancreas	PDAC	n.a.	G2	n.a.	1	1	1
LBp10	M68	Pancreas	PDAC	n.a.	G3	n.a.	1	1	1
LBp11	M50	Pancreas	PDAC	n.a.	G2	n.a.	2	2	4
LBp12	F69	Pancreas	PDAC	n.a.	G3	n.a.	0	0	0
LBp13	F54	Pancreas	PDAC	n.a.	G2	n.a.	1	1	1
LBp14	F61	Pancreas	PDAC	n.a.	G2	n.a.	0	0	0
LBp15	M65	Pancreas	PDAC	n.a.	G2	n.a.	1	1	1
LBp16	M61	Pancreas	PDAC	n.a.	G2	n.a.	0	0	0
LBp17	M59	Pancreas	PDAC	n.a.	G2	n.a.	2	1	2
LBp18	M60	Pancreas	PDAC	n.a.	G2	n.a.	0	0	0
LBp19	M46	Pancreas	PDAC	n.a.	G3	n.a.	0	0	0
LBp20	F/69	Pancreas	PDAC	n.a.	G3	n.a.	0	0	0
LBp21	M/88	Pancreas	PDAC	n.a.	G2	n.a.	2	1	2
LBp22	M77	Pancreas	PDAC	n.a.	G2	n.a.	1	1	1
LBp23	F54	Pancreas	PDAC	n.a.	G3	n.a.	0	0	0
LBp24	F70	Pancreas	PDAC	n.a.	G2	n.a.	1	1	0
LBp25	F61	Pancreas	PDAC	n.a.	G2	n.a.	1	2	2
LBp26	F79	Pancreas	PDAC	n.a.	G2	n.a.	2	2	4
LBp27	M67	Pancreas	PDAC	n.a.	G2	Gland-forming	0	0	0
LBp28	M49	Pancreas	PDAC	n.a.	G3	Gland-forming	2	1	2
LBp29	F64	Pancreas	PDAC	n.a.	G3	Solid	1	1	1
LBp30	F75	Pancreas	PDAC	n.a.	G2	Gland-forming	1	1	1
LBp31	M82	Pancreas	PDAC	n.a.	G3	Solid	0	0	0
LBp32	M75	Pancreas	PDAC	n.a.	G2	Gland-forming	1	1	1
LBp33	F65	Pancreas	PDAC	n.a.	G2	Gland-forming	0	0	0
LBp34	F82	Pancreas	PDAC	n.a.	G2	Gland-forming	0	0	0

PDAC *: pancreatic ductal adenocarcinoma* ***CCA*** *: cholangiocarcinoma;* ***n.a.*** *not assessed*
